# Frequency of interpersonal discrimination experiences – differences between inpatient and outpatient care and associations with delayed and forgone care

**DOI:** 10.1186/s12939-025-02512-4

**Published:** 2025-05-17

**Authors:** Olaf von dem Knesebeck, Demet Dingoyan, Anna Makowski, Jens Klein

**Affiliations:** Institute of Medical Sociology, University Medical Center Hamburg Eppendorf, Martinistr. 52, 20246 Hamburg, Germany

**Keywords:** Interpersonal discrimination, Inpatient care, Outpatient care, Unmet need, Population survey, Germany

## Abstract

**Background:**

While the number of studies investigating the magnitude, reasons, and possible consequences of perceived discrimination in health care is growing, many of these studies do not differentiate between structural and interpersonal discrimination in health care. The latter rests upon stereotypes and takes place in direct interactions between the health care provider and the patient. In this study, we explore the frequency and main reasons of different interpersonal discrimination experiences in inpatient and outpatient care in Germany and associations of discrimination with delayed and forgone care.

**Methods:**

Analyses were based on an online survey among the adult population in Germany (*N* = 3,246). A modified version of the Everyday Discrimination Scale was used to assess interpersonal discrimination experiences in inpatient and outpatient care. For each of the experiences, the main reason(s) for discrimination was ascertained. Indicators of delayed and forgone care referred to necessary doctor visits in the last 12 months.

**Results:**

Analyses revealed that two thirds (66%) of the respondents reported at least one of five experiences in inpatient or outpatient care. The experience that people in health care acted as if they were better than oneself was reported most frequently (41.3% in outpatient care and 27.7% in inpatient care). All discrimination experiences were significantly more frequent in outpatient care than in inpatient care. Age and health insurance were the most frequently reported reasons for the discrimination experiences. There was a significant association of the frequency of interpersonal discrimination experiences with delayed and forgone care.

**Conclusions:**

Experiences of interpersonal discrimination in health care were a frequent phenomenon in Germany and were significantly associated with unmet need. Such experiences must be considered an important issue for public health. Possible interventions to tackle discrimination include measures to reduce stereotypes and the abolition of the dual structure of statutory and private health insurance.

## Background

Discrimination can be defined as a behavioural pattern that reinforces stereotypes and disadvantages individuals or groups by differential treatment [1]. It can take place in interpersonal interactions or can be manifested in organizational and structural conditions like policies, regulations, or constitutional practices. Discrimination refers to labelled human differences that can be related to age, socio-economic status (SES), race/ethnicity, sex/gender, disability or specific diseases. Individuals who report having been discriminated often show multiple adverse health outcomes [2, 3].

Experiences of discrimination can occur in different contexts, including the health care system. In this regard, a study conducted in 30 European countries showed that 7% of the participants felt discriminated in a primary care practice in the last 12 months, with a range of 1.4–12.8% between the countries [4]. In a study from the U.S., 21% of the adult respondents indicated that they had experienced discrimination in the health care system [5]. In a recent survey in Germany, 26.6% of the respondents reported discrimination in health care [6]. In this survey, health issues or disability were the most frequently reported reasons for discrimination, followed by age and SES, whereas discrimination due to racism and sex/gender was less frequent. Discrimination experiences can interfere with acceptability of health services and therefore, can act as an access barrier that affects illness behaviour. In this regard, experiences of discrimination in health care have been found to be associated with delayed and forgone care in various countries like Germany [7], France [8], Sweden [9], and the U.S [10, 11]. Similar to experiences of discrimination, delayed and forgone care are socially patterned as studies have shown disparities in prevalence according to indicators of social inequalities [12].

While the number of studies investigating the magnitude, reasons, and possible consequences of perceived discrimination in health care is growing, some issues are not sufficiently clear. In this regard, many studies do not differentiate between structural and interpersonal discrimination in health care. While the former is defined by institutional practices and policies that work to the disadvantage of certain individuals or patient groups, the latter is taking place in direct interactions between the health care provider and the patient [13]. Moreover, in many studies it is only asked whether people had ever been discriminated against [4, 6–8] but the frequency of discrimination experiences is rarely assessed [5, 14]. Relatedly, measures used often are quite general as they do not ask for different (concrete) discrimination experiences. Finally, most studies generally refer to discrimination in medical care or in the health care system and do not consider different medical or health care settings [15] like inpatient and outpatient care. It can be expected that varying structures and processes in inpatient and outpatient care may result in different levels of interpersonal discrimination.

With the present study, we would like to pick up these issues by using a modified version of the Everyday Discrimination Scale [16] to explore the frequency of different interpersonal discrimination experiences in inpatient and outpatient care in Germany. More specifically, the following research questions will be addressed: (1) How often do people report different experiences of interpersonal discrimination in health care? (2) Are there differences in the reported frequency of discrimination experiences between inpatient and outpatient care? (3) What are the main reasons for the different discrimination experiences in inpatient and outpatient care? (4) Are experiences of interpersonal discrimination in inpatient and outpatient care associated with delayed and forgone care?

## Methods

### Study design and sample

Analyses are based on a cross-sectional online survey on discrimination in health care in Germany. The survey was conducted by a social research institute (forsa) in February and March 2024. An adult population sample (age 18 + years) was randomly drawn from a panel which was recruited offline via telephone. To this end, a dual-frame approach was applied that included landline as well as mobile phone numbers. The panel is a population-based, representative sample of the adult population living in Germany that is continuously refreshed and consisted of about 150,000 people at the time of data collection. 8,025 individuals who reported to use the internet were randomly selected from the panel and invited to participate in the present survey via email. After two reminders, *N* = 3,246 individuals participated. To explore a variety of reasons for the different discrimination experiences in inpatient and outpatient care, we aimed at a fairly large sample size of about 3,200. Sample was weighted by sex, age, federal state, and education using the iterative proportional fitting approach [17]. To this end, the official statistics provided by the Federal Statistical Office of Germany were used [18]. Thus, the sample adequately represents the adult population in Germany regarding these socio-demographic characteristics. The survey was approved by the Local Psychological Ethics Committee at the Center for Psychosocial Medicine, University Medical Center Hamburg (No. LPEK-0719). All research was performed in accordance with relevant guidelines/regulations and the Declaration of Helsinki.

### Measures

Assessment of the frequency of interpersonal discrimination experiences in inpatient and outpatient care was based on the Everyday Discrimination Scale [16] originally consisting of nine items. The scale was cut and modified for the specific context of discrimination in health care [19]. Five of the nine items were used: The two items regarding courtesy and respect were combined: “you were treated with less courtesy or respect than others”. The original item “you received poorer service than others in restaurants and stores” was rephrased into “you received a poorer medical treatment than others”. Three items were used unmodified (“people acted as if you are not smart”, “people acted as if they are afraid of you”, and “people acted as if they are better than you”) and three items were omitted (“people acted as if you are dishonest”, “you are called names or insulted”, and “you are threatened or harassed”) because we considered them less relevant for the context of health care. The items were introduced by the statement: “In the following, we will ask you some questions about your experiences with health care staff.” Afterwards, it was asked: “How often have any of the following things happened to you in a medical practice?” and “How often have any of the following things happened to you in a hospital?”. Participants who stated that they have not visited any in- or outpatient facilities were excluded. Response categories were “never”, “rarely”, “sometimes”, “often”, and “very often”. Sum scales were calculated using the situational coding approach [20], i.e. we dichotomised each item to ‘never’= 0 and ‘ever’ (collapsing those reporting ‘rarely’ or more often into one category) = 1. Items were then summed (range: 0–5) to assess the total number of situations ever experienced in a medical practice or a hospital.

For each of the items, the main reason(s) for the discrimination experience was ascertained when the respective situation was experienced rarely or more often. In this case, respondents could choose one or more reasons from a list covering: origin/name/migration history, religion, language problems, colour of skin, income, education, occupation, unemployment, sex, age, disability, mental illness/addiction, weight, appearance, and health insurance. The latter was included as a possible reason because there is a dual structure of statutory and private health insurance in Germany and there are incentives for the preferential treatment of privately insured patients. For the analyses, origin/name/migration history, religion, language problems, and colour of skin were categorized as “racism”. We decided to consider religious discrimination as racism because this form of discrimination is often associated with islamophobia in Germany. Income, education, occupation, and unemployment were summarized as “socio-economic status” (SES).

To measure delayed care, it was asked whether it happened in the last 12 months that the respondents have delayed a necessary visit to a doctor (yes/no). The respective question regarding forgone care was: “In the last 12 months, did it happen that you have forgone a necessary visit to a doctor?” (yes/no). Both items were based on questions used in the European Health Interview Survey [21]. If respondents indicated, that there was no need for treatment in the past 12 months, they were excluded from analyses regarding delayed and forgone care.

Sex, age, migration history, education, income, and health insurance were considered as covariates in the multiple logistic regression analyses (please see below). Age was divided into three groups (18–40, 41–60, and 61 + years). In terms of education, the CASMIN educational classification was used [22]. The nine original CASMIN-levels were merged into three educational groups. Monthly net household income was equalized to consider household size and composition. The variable was further divided into tertiles. Regarding migration history, respondents were also categorized into three groups: those who have immigrated themselves (1st generation migrants); those who were born in Germany but whose parents (one or both) have immigrated (2nd generation migrants), and those without a migration history. Finally, respondents were asked whether they have a private or a statutory health insurance.

### Analyses

Frequencies of the five different experiences of interpersonal discrimination in health care were analysed. To explore differences between inpatient and outpatient care, chi square tests (single items) and Mann-Whitney-U tests (sum scales) were used. Subsequently, frequencies of the nine reasons for the different discrimination experiences were analysed. Associations between interpersonal discrimination experiences (sum scales) and delayed/forgone care were explored via crosstabs and multiple logistic regression analyses. For the latter, sum scales and covariates (sex, age, migration history, education, income, and health insurance) were introduced separately (unadjusted, model 1) as well as simultaneously (fully adjusted, model 2). Odds ratios, 95%-confidence intervals, significances, and explained variances (Nagelkerke’s R^2^) are documented. Statistical procedures were performed with the statistical program package SPSS 29 [23].

## Results

Sample description regarding socio-demographic characteristics, health insurance as well delayed and forgone care is documented in Table 1. Almost 50% of the respondents reported that they delayed necessary care in the last 12 months and about one third reported forgone care. There was a significant correlation between delayed and forgone care (*r* = 0.60, *p* < 0.001).

**Table 1:** Sample description (*N* = 3,246)^a^

Variable	*n* (%)
Age (years) *(0)*	
18–40	1,078 (33.2)
41–60	1,103 (34.0)
> 60	1,065 (32.8)
Sex *(0)*	
Female	1,661 (51.2)
Male	1,585 (48.8)
Migration history *(0)*	
No	2,451 (75.5)
1st generation	289 (8.9)
2nd generation	506 (15.6)
Income *(377)*	
High	952 (33.2)
Intermediate	907 (31.6)
Low	1,010 (35.2)
Education *(31)*	
High	860 (26.8)
Intermediate	1,021 (31.8)
Low	1,333 (41.4)
Health insurance *(8)*	
Statutory	2,870 (88.6)
Private	368 (11.4)
Delayed care (331^b^)	
Yes	1,424 (48.9)
No	1,491 (51.1)
Forgone care (348^b^)	
Yes	972 (33.5)
No	1,926 (66.5)

^a^weighted; number of missing cases in brackets/italics; ^b^including respondents who did not need medical care in the last 12 months

In terms of the different experiences of interpersonal discrimination in health care (Table 2), respondents most frequently reported that health care staff acted as if they are better (41.3% in outpatient care and 27.7% in inpatient care), while experiences of people acting as if they are afraid were least common (4.8%-3.2%). When respondents report having been discriminated, this mostly happened rarely or sometimes while a minority made these experiences often or very often. All discrimination experiences were significantly more frequent in outpatient care. Sum scales also differed significantly.

**Table 2:** Frequency of different experiences of interpersonal discrimination in health care (%)

	Outpatient care (*n* = 3,219)^a^	Inpatient care (*n* = 2,792)^b^	*p* (χ^2^)
*You were treated with less courtesy or respect than others.*			< 0.001
Never Rarely Sometimes Often Very often	72.9 17.1 6.9 2.0 1.0	78.3 13.8 5.6 1.7 0.7	
*You received a poorer medical treatment than others.*			< 0.001
Never Rarely Sometimes Often Very often	74.2 15.6 7.8 1.7 0.7	80.6 11.3 5.8 1.5 0.7	
*People acted as if you are not smart.*			< 0.001
Never Rarely Sometimes Often Very often	74.0 15.5 7.9 2.1 0.4	81.9 10.9 5.3 1.3 0.6	
*People acted as if they are afraid of you.*			< 0.001
Never Rarely Sometimes Often Very often	95.2 2.9 1.4 0.4 0.1	96.8 2.6 0.4 0.2 0.1	
*People acted as if they are better than you.*			< 0.001
Never Rarely Sometimes Often Very often	58.7 23.2 13.2 3.5 1.4	72.3 16.4 7.8 2.5 1.0	
	p (Mann-Whitney-U)		
*Sum scale*,* situation-based coding * *(Range 0–5; Mean (SD))*	1.20 (1.29)	0.86 (1.26)	< 0.001

^a^ excluding respondents who have never been in a medical practice; ^b^ excluding respondents who have never been in a hospital

Additional analyses (not shown in Table 2) revealed that 59.9% of the respondents reported at least one of the five experiences in outpatient care (rarely or more often). Respective number for inpatient care was 41.8% and two thirds (66%) of the respondents had at least one experience in inpatient or outpatient care.

About 24% of the respondents who experienced less courtesy or respect in outpatient care at least rarely, reported that this experience was due to their health insurance (Table 3, upper part). Also age was a frequently reported reason for this experience. In terms of poorer treatment in outpatient care, more than 50% of the respondents indicated that this experience was due to their health insurance. The most frequently mentioned reason for having been treated as one was not smart was age (34.1%), while it was appearance (24.9%) for having been treated as if the health care staff in a medical practice was afraid. Age (23.8%) was most often reported as the reason for having been treated as if someone was better than oneself. The pattern of reasons for discrimination experiences in inpatient care was similar (lower part of Table 3).

**Table 3:** Reasons for different experiences of interpersonal discrimination in health care (%)^a^

	Outpatient care				
	Less courtesy/respect (*n* = 849)	Poorer treatment (*n* = 696)	Not smart (*n* = 825)	Afraid of you (*n* = 152)	Better than you (*n* = 1,303)
Racism^b^ Sex Age Disability Mental illness/addiction Weight Appearance Socio-economic status^c^ Health insurance	5.0 5.5 23.4 2.6 3.7 11.1 13.0 7.2 **24.1**	3.6 4.5 15.0 1.8 4.2 8.6 7.0 7.9 **52.1**	6.4 9.0 **34.1** 4.1 5.9 11.8 13.2 12.3 10.7	18.3 3.3 9.2 7.2 8.1 9.9 **24.9** 10.7 2.8	5.9 6.0 **23.8** 2.5 3.6 12.1 14.5 20.2 23.4
	Inpatient care				
	Less courtesy/respect (*n* = 571)	Poorer treatment (*n* = 447)	Not smart (*n* = 480)	Afraid of you (*n* = 86)	Better than you (*n* = 730)
Racism^b^ Sex Age Disability Mental illness/addiction Weight Appearance Socio-economic status^c^ Health insurance	6.5 5.4 25.0 3.8 5.2 11.4 11.2 8.3 **28.9**	4.7 2.1 14.8 1.8 4.4 7.8 7.3 7.2 **59.1**	7.3 7.6 **34.9** 5.7 7.9 12.2 14.5 15.7 14.8	15.7 8.3 **19.9** 14.8 6.1 4.4 16.6 11.9 13.7	6.6 6.7 **26.5** 4.5 5.0 12.5 14.3 20.3 26.3

^a^ multiple responses were possible, percentages do not necessarily add up to 100% due to other reasons and missings (don’t know/no answer); ^b^ origin/name/migration history, religion, language problems, and colour of skin; ^c^ income, education, occupation, and unemployment; most frequently mentioned reasons are bold

Interpersonal discrimination experiences in inpatient and outpatient care (sum scales) were significantly associated with delayed and forgone care (Table 4). This association tended to be gradual, i.e. the more discrimination experiences the more often respondents reported delayed and forgone care.

**Table 4:** Association between interpersonal discrimination experiences (sum scales) and proportions of delayed /forgone care (*n* = 2,531-2,909)

	Delayed care (%)	Forgone care (%)
Discrimination in outpatient care		
No experiences		
1 experience *≥* 2 experiences	38.6 47.2 62.2	24.6 29.8 46.8
p (χ2)	< 0.001	< 0.001
Discrimination in inpatient care		
No experiences 1 experience *≥* 2 experiences	43.5 50.3 64.4	28.9 31.0 50.8
p (χ2)	< 0.001	< 0.001

Multiple logistic regression analyses shows that the associations remained significant after adjustment of the covariates sex, age, migration history, education, income, and health insurance (Table 5, fully adjusted models). Accordingly, respondents with two or more discrimination experiences in inpatient or outpatient care were 1.6 to 2.0 times more likely to report delayed or forgone care. Significantly increased odds ratios for delayed or forgone care were also found for female sex, lower age, and low income, while significant associations with low education (delayed care) and statutory health insurance (delayed and forgone care) were restricted to the unadjusted model.

**Table 5:** Logistic regression analyses: odds ratios, (95% confidence intervals), significances (*n* = 2,264-2,274)

	Delayed care		Forgone care	
	Unadjusted (model 1)	Fully adjusted (model 2)	Unadjusted (model 1)	Fully adjusted (model 2)
Discrimination (sum scales) Outpatient				
1 experience *≥* 2 experiences	1.42 (1.18–1.71)*** 2.61 (2.19–3.10)***	1.23 (0.98–1.53) 1.83 (1.44–2.31)***	1.30 (1.06–1.60)* 2.70 (2.25–3.25)***	1.21 (0.95–1.55) 2.03 (1.59–2.58)***
Inpatient				
1 experience *≥* 2 experiences	1.32 (1.07–1.62)* 2.35 (1.93–2.87)***	1.11 (0.88–1.41) 1.62 (1.27–2.07)***	1.11 (0.88–1.39) 2.54 (2.09–3.10)***	0.89 (0.69–1.15) 1.61 (1.26–2.05)***
Sex				
Female	1.68 (145–194)***	1.59 (1.33–1.89)***	1.40 (1.20–1.64)***	1.32 (1.10–1.59)**
Age 18–40 years 41–60 years	1.85 (1.54–2.21)*** 1.64 1.38-(1.97)***	1.56 (1.22-2.00)*** 1.53 (1.24–1.90)***	1.66 (1.36–2.02)*** 1.86 (1.53–2.25)***	1.52 (1.16–1.97)** 1.82 (1.45–2.28)***
Migration history				
1st generation 2nd generation	1.00 (0.77–1.30) 1.14 (0.93–1.39)	1.00 (0.73–1.37) 1.02 (0.80–1.31)	1.24 (0.95–1.61) 0.95 (0.76–1.19)	1.25 (0.91–1.71) 0.93 (0.72–1.20)
Education				
low intermediate	0.68 (0.57–0.82)*** 0.93 (0.78–1.12)	0.83 (0.65–1.06) 0.93 (0.75–1.16)	0.94 (0.78–1.14) 1.13 (0.94–1.35)	1.02 (0.79–1.32) 1.11 (0.88–1.39)
Income				
low intermediate	1.55 (1.28–187)*** 1.08 (0.89–1.31)	1.49 (1.20–1.86)*** 1.15 (0.93–1.43)	1.42 (1.16–1.72)*** 1.04 (0.85–1.28)	1.27 (1.01–1.60)* 1.04 (0.83–1.31)
Health insurance				
Statutory	1.34 (1.06–1.69)*	1.25 (0.94–1.66)	1.36 (1.05–1.75)*	1.34 (0.98–1.85)
Nagelkerke’s R^2^		0.11		0.10

Reference categories: no discrimination experiences, male, 61 years +, no migration history, high education, high income, private health insurance; * *p* < 0.05, ** *p* < 0.01, *** *p* < 0.001

## Discussion

### Summary and interpretation

Based on a population survey, the present study found that experiences of interpersonal discrimination in health care are a frequent phenomenon in Germany. Two thirds (66%) of the respondents reported at least one of five experiences in inpatient or outpatient care. The experience that people in health care acted as if they were better than oneself was reported most frequently (41.3% in outpatient care and 27.7% in inpatient care), while only a few respondents experienced that people acted as if they were afraid of them (4.8%-3.2%). All discrimination experiences were significantly more frequent in outpatient care than in inpatient care. Age and health insurance were the most frequently reported reasons for the discrimination experiences in outpatient and inpatient care. There was a significant association between frequency of interpersonal discrimination experiences and delayed/forgone care.

Compared to a previous study in which 26.6% of the respondents reported discrimination in health care in Germany [6], frequency of discrimination experiences in the present study was considerably higher. This discrepancy is probably due to different measurement approaches. While the previous study applied a general one item-assessment, we asked for the frequency of five concrete experiences. This indicates that a crude assessment may underestimate discrimination in health care. In terms of the frequency of the different experiences, our results are comparable with a study among African American patients [19]. The authors also found the experience that people in health care acted as if they were afraid of the respondents to be least frequent whereas being treated as inferior was among the most frequent experiences.

To the best of our knowledge, this is the first study comparing discrimination in inpatient and outpatient care. The higher reported frequency in outpatient care may be explained by the fact that people more often visit primary-care and specialist physicians than they stay in hospitals. Access problems in outpatient care e.g. due to waiting times for an appointment may also play a role for the larger frequency of reported discrimination [24]. Moreover, treatment is expected to be more standardized in inpatient care (e.g. by the use of guidelines or standard operating procedures) than in outpatient care, leaving potentially less room for variations in behaviour and for interpersonal discrimination. In addition, the duration and type of interaction may play a role. Furthermore, a team of health care professionals is involved in inpatient care which implies a higher level of supervision and mutual control.

Although reported reasons for discrimination differed according to the five experiences under study, there were two reasons that stood out: age and health insurance. Age was also an important reason in a previous German study [6], while in the U.S., other reasons like race/ethnicity or SES were mentioned more frequently [5]. Our results show that age was the main reason among those who experienced being treated as if they were not smart or as if someone was better than they are. A previous study indicated that young people more often report age as a reason for discrimination experiences than older age groups [6]. Thus, it can be expected that people feel discriminated on the basis of a perception of them as being young or too young [25].

Type of health insurance was the main reason for reporting that one was treated with less courtesy or respect than others or received a poorer medical treatment than others. In Germany, there is a dual structure of statutory and private health insurance. A private insurance can only be chosen by people with an income over a certain limit, self-employed, and public servants. Different physician reimbursement rates create incentives for the preferential treatment of privately insured patients. Thus, the system in a way discriminates statutorily insured patients [24] which is reflected in the present data. Insurance-based discrimination was also reported in other countries like the U.S [26]. It can be expected that such discrimination is more pronounced in the U.S. where many people are uninsured while health insurance generally is compulsory for everyone living in Germany.

Finally, our analyses showed that respondents with two or more discrimination experiences in inpatient or outpatient care were 1.6 to 2.0 times more likely to report delayed or forgone care in the last 12 months while the likelihood of those who reported only one experience was not significantly increased. For these analyses we used a sum scale based on the situational coding approach for the Everyday Discrimination Scale [20]. Delayed and forgone care are commonly used as indicators for unmet need [12, 27] or access to health care [26]. Our results confirm that people may delay or forgo care because of repeated experiences of discrimination. Referring to the concept of access proposed by Penchansky and Thomas [28], discrimination experiences can interfere with acceptability of health services and act as an access barrier [8].

### Limitations

When evaluating the reported findings a couple of limitations should be considered. Analyses were based on an online survey. Although a random sample was drawn from a panel which was recruited offline, only those who use the internet were included. Access barriers for using the internet and varying internet user behaviours may contribute to a selection bias. In this regard, it is likely that certain groups (e.g. very old people or individuals with severe health problems and limitations) are underrepresented in the sample. Moreover, only about 40.4% of the invited persons participated.

Furthermore, the questionnaire used was in German. Thus, people with problems in reading or writing in German are underrepresented and experiences of discrimination due to language problems are likely to be underestimated. We used a modified version of the Everyday Discrimination Scale [16], a widely used measure of self-reported discrimination experiences. Although similar modifications have been used for the specific context of health care [19], our scale cannot be considered validated as only few studies applied a translated version of the Everyday Discrimination Scale in a health or health care related context in Germany [29, 30].

## Conclusions

Experiences of interpersonal discrimination in health care were a frequent phenomenon in Germany. Prevalence was higher in outpatient than in inpatient care. Dismissive behaviour was most frequently reported. Age and type of health insurance overall were the most prevalent reasons. Experiences of discrimination were significantly associated with unmet need. Interpersonal discrimination in health care is an important issue for public health and health services research. Possible interventions to tackle discrimination include measures to reduce stereotypes which should be integrated in medical education and in continuing medical training. Also, abolition of the dual structure of statutory and private health insurance would certainly reduce discrimination as reimbursement rates create incentives for the preferential treatment of privately insured patients.

## Data Availability

The dataset used is available from the corresponding author on reasonable request.
